# Annotations

**Published:** 1908-10-17

**Authors:** 


### ANNOTATIONS.

### Compulsory Amputation.

Under the striking headline "Court Orders Amputation," our esteemed contemporary, *The Medical Record* of New York, publishes a remarkable paragraph, which deserves quotation in its entirety: "Because of the opposition of his parents to the operation, surgeons of the County Hospital of Chicago were compelled to obtain an order from the Court directing the amputation of the arm of a fourteen-year-old boy recently. Gangrene following a fracture made the operation necessary, but neither the boy nor his parents would consent." We should not like to question the accuracy of this paragraph; but as it stands it is a little startling to British ears, accustomed to a large degree of personal freedom, and impatient of official interference in matters affecting the individual as opposed to the collective health. However important it may be to secure obedience to medical orders, it seems doubtful whether—even in the ideal republic—the enforcement of a surgical operation should properly come within the jurisdiction of a court of law. The action of the Chicago Court in this instance, although certainly in the best interests of the fourteen-year-old boy, savours somewhat of a violation of the liberty of the subject. To perform an immediate operation, without waiting to obtain permission, upon a hospital patient whose condition is one of extreme urgency, is one thing; but to resort to compulsion (whether legally sanctioned or otherwise) when persuasion has failed is quite another. For his own sake, as well as in the public interest, an ignorant person can be isolated against his will if he breaks the law, or if he becomes afflicted with mental or infectious disease, and education and vaccination also can (with more or less success) be thrust upon him, but the time scarcely seems ripe for compulsory operations.

### Colour-blindness.

The subject of colour-blindness is, of course, one which has more aspects than the purely scientific. From time to time this curious deficiency assumes an air of great practical importance in the eyes of the general public, more particularly in connection with the dangers of travel by land and sea. But there are other occupations besides those of engine-drivers, signalmen, and navigators in which a perfect colour sense is of the greatest importance. The illustrated address on this subject lately given by Dr. F. W. Edridge Green before the physiological section of the British Association deserves, therefore, some measure of public attention, and is of considerable interest to medical men. After dealing at length with the nature and varieties of colour-blindness, Dr. Edridge Green criticised very severely the familiar wool test, associated with the name of Holmgren, which is undoubtedly the most popular test for colour-blindness in this country. He stated that it is now generally recognised that the wool test is quite inefficient, and that nearly every civilised country, except our own, has adopted the lantern test. Apparently Dr. Edridge Green pointed out the necessity for the lantern test nearly 20 years ago, as well as the necessity for varying the intensity of the light and using the names of colours. The first requirement of a test for colour-blindness is that colour names should be used and that both the examiner and examinee should employ and understand the use of the colour names—red, yellow, green, and blue. He says that no test that ignores colour names can be efficient. Nothing shows the value of colour names better than an examination with his lantern of certain educated colour-blind persons who have succeeded in passing other well-known tests with ease. They call red "green" and green "red," and apply either term to yellow, thus not only proving their colour-blindness, but also showing how absolutely unfit they are to act as engine-drivers or look-outs. It certainly seems as though the greatest maritime and commercial nation is behind her rivals in the methods of safeguarding traffic from the dangers due to colour-blindness.

### The Responsibility of the Surgeon.

If Professor Emile Forgue, who presided over the twenty-first French Congress of Surgery in Paris last week, has been correctly reported, he has laid down a canon of surgical conduct which, while true enough in a way, is yet capable of misconstruction. He was addressing his audience on the social importance of the surgeon's art, and the readiness of the members of the profession to assume all right and necessary responsibilities, and he claimed that "the chief concern of the surgeon is the maintenance of a steady progress in his art." Now no one will deny that this ideal is one which every surgeon should, and probably does, keep steadfastly before him; but in actual practice the first concern of the surgeon, or any other medical practitioner, must be the interests of the particular patient with whom he is dealing. We do not suggest for a second that Professor Forgue is not preaching this in effect, for he would rightly argue that progress in his art is what the surgeon must ever strive after in order to do his duty to his patients, present and prospective. But to put the matter in this way is to risk the revival of an old and galling accusation of the laity against the medical profession—that of treating patients too much as museum specimens, and too little as human beings. This reproach has, we must admit, very occasionally had a substratum of justification; and we think it is to the credit of profession and public alike that so little has been heard of this taunt during the past few years. There is nothing so repugnant to the ordinary Briton as the idea that the artistic possibilities of operations are of more importance to the surgeon than their probable benefit to those who undergo them, and we believe we are correct in asserting that nowhere in the world are the members of our profession less open to such a suspicion than in this country. With this reservation we are entirely with the President of the Congress in supporting the claims of surgery to equitable recognition in accordance with its high responsibilities.

